# Role of Kynurenine and Its Derivatives in Liver Diseases: Recent Advances and Future Clinical Perspectives

**DOI:** 10.3390/ijms26030968

**Published:** 2025-01-24

**Authors:** Qiwen Tan, Shenghe Deng, Lijuan Xiong

**Affiliations:** 1 Department of Infectious Disease, Union Hospital, Tongji Medical College, Huazhong University of Science and Technology, Wuhan 430022, China; 15703054626@163.com; 2 Center for Liver Transplantation, Union Hospital, Tongji Medical College, Huazhong University of Science and Technology, Wuhan 430022, China; 3 Department of Nosocomial Infection Management, Union Hospital, Tongji Medical College, Huazhong University of Science and Technology, Wuhan 430022, China

**Keywords:** tryptophan metabolite, kynurenine, kynurenine pathway, liver disease

## Abstract

Liver health is integral to overall human well-being and the pathogenesis of various diseases. In recent years, kynurenine and its derivatives have gradually been recognized for their involvement in various pathophysiological processes, especially in the regulation of liver diseases, such as acute liver injury, non-alcoholic fatty liver disease, cirrhosis, and liver cancer. Kynurenine and its derivatives are derived from tryptophan, which is broken down by the enzymes indoleamine 2,3-dioxygenase (IDO) and tryptophan 2,3-dioxygenase (TDO), converting the essential amino acid tryptophan into kynurenine (KYN) and other downstream metabolites, such as kynurenic acid (KYNA), 3-hydroxykynurenine (3-HK), xanthurenic acid (XA), and quinolinic acid (QA). In liver diseases, kynurenine and its derivatives can promote the activity of the transcription factor aryl hydrocarbon receptor (AhR), suppress T cell activity for immune modulation, inhibit the activation of inflammatory signaling pathways, such as NF-κB for anti-inflammatory effects, and inhibit the activation of hepatic stellate cells to slow down fibrosis progression. Additionally, kynurenine and other downstream metabolites can influence the progression of liver diseases by modulating the gut microbiota. Therefore, in this review, we summarize and explore the mechanisms by which kynurenine and its derivatives regulate liver diseases to help develop new diagnostic or prognostic biomarkers and effective therapies targeting the kynurenine pathway for liver disease treatment.

## 1. Introduction

Over 2 million people die from liver diseases worldwide each year, accounting for about 4% of global deaths, with alcoholic liver disease, cirrhosis, and liver cancer being the most common causes, severely threatening human health [1]. Chronic liver disease affects an estimated 1.5 billion individuals globally, with its prevalence having risen by 13% since 2000, thereby imposing a significant burden on populations worldwide [2]. Chronic liver disease develops from liver injury, with common pathological features of liver injury including hepatocyte degeneration, necrosis, and apoptosis [3]. Hepatocyte death triggers subsequent inflammation, leading to excessive deposition of extracellular matrix proteins, resulting in hepatitis and liver fibrosis, and eventually progressing to cirrhosis and hepatocellular carcinoma [4]. Currently, therapeutic interventions for liver diseases are rapidly advancing, aiming to delay or even reverse the progression of liver diseases.

In recent years, kynurenine and its derivatives have exhibited anti-inflammatory, immunoregulatory, and pro-apoptotic properties, contributing to the regulation of liver lipid metabolism, inflammation, and fibrosis. The kynurenine pathway is the main pathway of tryptophan metabolism. In the body, 2,3-dioxygenase (TDO) and two indoleamine 2,3-dioxygenase isoforms (IDO-1 and IDO-2) oxidize tryptophan into kynurenine and its series of derivatives [5]. Kynurenine (Kyn) has immunoregulatory effects, promoting the stagnation or apoptosis of effector T cells [6] and inducing the conversion of naive CD4+ T cells into immunosuppressive regulatory T cells (Treg) [7]. Ogiso et al. discovered that the lack of the Ido1 gene exacerbated CCl4-induced liver fibrosis in a CCl4-induced liver injury model [8]. In primary liver cancer, the kynurenine pathway (KP) enables tumor cells to escape immune surveillance [9], promoting proliferation and metastasis, as well as suppressing local immune cell populations [10], thereby facilitating tumor growth. Recent studies have highlighted the potential link between the kynurenine pathway and various liver diseases, including acute and chronic liver injury [11,12], non-alcoholic fatty liver disease [13], and liver cancer [14].

To further clarify the regulatory effects of kynurenine and its derivatives on liver diseases, this article comprehensively introduces the synthesis, metabolism, bioactivity, and mechanisms of action in liver disease regulation, and the clinical challenges faced, as well as discusses how to expedite the clinical translation and application of kynurenine and its derivatives. The aim of this review is to bridge the gap between clinical treatment of liver diseases and basic experimental to clinical translation, providing theoretical reference for the development of kynurenine and its derivatives in the field of liver disease treatment.

## 2. Synthesis, Metabolism, and Biological Functions of Kynurenine

### 2.1. Synthesis and Metabolism of Kynurenine

Kynurenic acid (KYNA) was first isolated by Justus von Liebig from the urine of dogs fed with tryptophan. Subsequently, Kotake and Iwao isolated kynurenine as the main metabolic product of tryptophan (TRP) breakdown through the kynurenine pathway [15].Tryptophan can produce various metabolites through direct or indirect action by the gut microbiota and then be transported by transport proteins to intestinal epithelial cells, reaching the liver via the enterohepatic circulation, influencing liver function and metabolism [16]. In the gut, tryptophan mainly follows three metabolic pathways, including the kynurenine pathway [17], serotonin metabolism pathway [18], and indole and its derivative metabolism pathway [19].

**Figure 1:**
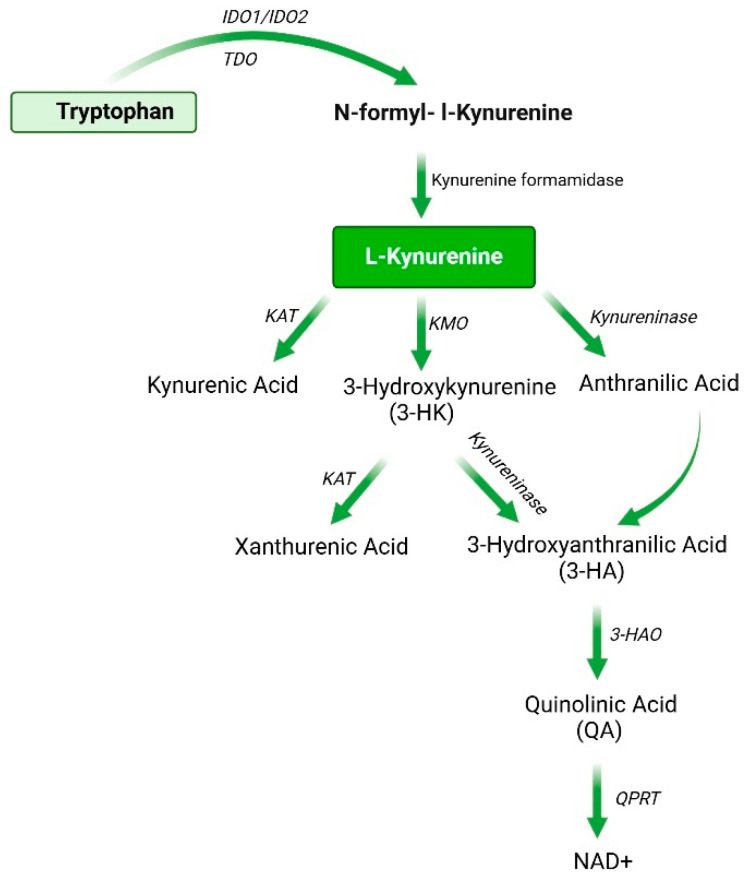
Overview of the kynurenine pathway (KP). This figure shows the main metabolites and enzymes of the KP. Abbreviations: NAD+, nicotinamide adenine dinucleotide; IDO, indoleamine 2,3-dioxygenase; TDO, Tryptophan 2,3-dioxygenase; KMO, kynurenine 3-monooxygenase; KAT, kynurenine aminotransferases; KMO, kynurenine 3-monooxygenase; 3-HAO, 3-hydroxyanthranilate 3,4-dioxygenase; QPRT, quinolinic acid phosphoribosyl transferase.

More than 95% of tryptophan is metabolized via the kynurenine pathway, primarily by the rate-limiting enzymes indoleamine 2,3-dioxygenases (IDO1 and IDO2), kynurenine formamidase, kynurenine aminotransferase (KAT), kynurenine 3-monooxygenase (KMO), 3-hydroxyanthranilic acid oxidase (3-HAO), quinolinic acid phosphoribosyl transferase (QPRT), and tryptophan-2,3-dioxygenase (TDO) [20]. Studies have shown that tryptophan 2,3-dioxygenase (TDO) is predominantly expressed in the liver, especially in hepatocytes [21]. Furthermore, the expression level of Tryptophan 2,3-dioxygenase (TDO) is regulated by factors such as L-tryptophan (Trp), corticosteroids, and hypoxic conditions [22,23]. IDO1 is widely expressed in various stem cells, such as bone marrow mesenchymal stem cells (BMSCs) and umbilical cord tissue-derived mesenchymal stromal cells (UC-MSC), and multiple immune cells, including dendritic cells, macrophages, and neutrophils [24,25,26]. It plays a critical role in immune regulation and liver repair and regeneration [27]. The expression of IDO1 is regulated by various factors, such as interferon-γ (IFN-γ), tumor necrosis factor-alpha (TNF-α), and stimulation by other cytokines [28]. Therefore, the activity of IDO1 is associated with immune responses, chronic inflammation, tumor immune escape, and various other pathological processes. Additionally, the kynurenine pathway is tightly regulated under physiological conditions, generating various metabolites through the action of a series of enzymes (Figure 1), such as kynurenine, kynurenic acid (KYNA), 3-hydroxykynurenine (3-HK), anthranilic acid (QA), quinolinic acid (QA), and the NAD(P)/NAD(P)H [29].

**Figure 2:**
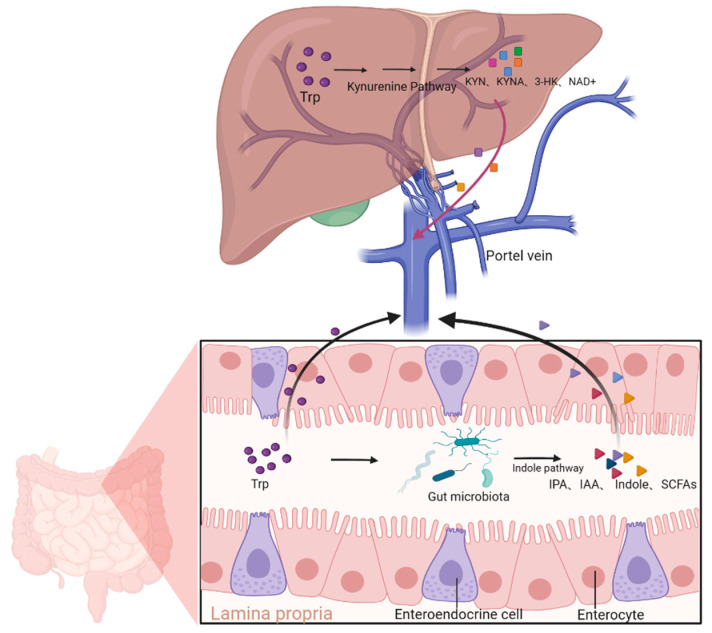
The interaction between the gut and liver. Tryptophan is partially absorbed in the gut, while the rest is metabolized by gut microbiota through the indole pathway into various beneficial or harmful metabolites like IPA, IAA, indole, SCFAs, etc., which then enter the portal vein system and reach the liver, modulating the kynurenine pathway. On the other hand, kynurenine metabolites, such as KYN, KYNA, 3-HKK, IDO, etc., are also capable of returning to the gut to exert regulatory effects. Abbreviations: KYNA, kynurenic acid; NAD+, nicotinamide adenine dinucleotide; 3-HK, 3-hydrokynurenine; Trp, tryptophan; IPA, indole-3-propionic acid; IAA, indole-3-acetic acid; SCFAs, short-chain fatty acids.

Furthermore, kynurenine, as a critical intermediate in tryptophan metabolism, is closely linked to the metabolism and function of the gut microbiota. The kynurenine pathway (KP) plays an essential role in regulating the immune system, neuro-signaling, and oxidative stress. The interaction between the gut and liver is referred to as the “gut–liver axis”, which is a complex bidirectional connection based on the portal vein and involves various signaling pathways. Kynurenine metabolism in the liver is also regulated by the gut (Figure 2). Kynurenine is a key intermediate in tryptophan metabolism, and its metabolic pathway is closely associated with the metabolism and function of the gut microbiota. Tryptophan absorption primarily occurs at the apical membrane of intestinal epithelial cells, where it is transported into the cells via angiotensin-converting enzyme 2 (ACE2) and broad-spectrum neutral amino acid transporter 1 (B0AT1) [30]. Once absorbed by transport proteins in the intestinal epithelial cells, tryptophan (TRP) passes through the portal venous system and is converted to NAD+ in the liver via the kynurenine pathway [31]. The gut, as an important site for tryptophan metabolism, widely expresses IDO in intestinal epithelial cells and immune cells. Gut microbiota significantly modulates the activity of the host’s tryptophan metabolic pathway by secreting indole metabolites, short-chain fatty acids (SCFAs), and other signaling molecules [32].

The gut microbiota regulates the host’s kynurenine metabolism through multiple mechanisms, including direct regulation of IDO1 activity and indirect influence on tryptophan metabolic pathways. The gut microbiota can directly regulate the kynurenine metabolism. Zhao et al. found that in a colitis mouse model, the gut microbiota was found to regulate IDO-1 and KAT, leading to a significant increase in Kyn and kynurenic acid levels [33]. Additionally, gut microbiota can indirectly regulate the kynurenine pathway through metabolites derived from the indole pathway. Furthermore, recent studies have shown that in mice with dysbiosis, gut barrier dysfunction and tryptophan metabolic disorders lead to a significant increase in indole derivatives, which activate the colon aryl hydrocarbon receptor (AhR), promote the kynurenine pathway, and inhibit the serotonin pathway [34]. Similarly, Paydaş Hataysal et al. found that in patients with inflammatory bowel disease, serum levels of tryptophan, kynurenic acid, 3-hydroxykynurenine, and other kynurenine metabolites are lower [35]. Indole metabolites, such as indole-3-propionic acid and indole-lactic acid, are products of gut microbiota metabolism of tryptophan and can regulate IDO1 expression through the aryl hydrocarbon receptor (AhR) [36].

Kynurenine and its metabolites can also feedback to affect the composition and function of the gut microbiota. Kynurenine can also be metabolized in the gut to substances, such as 3-HK and KA, and kynurenine metabolites can indirectly alter the gut microbiota composition by affecting the local immune microenvironment. Kynurenic acid is an antimicrobial substance in the intestinal fluid, first isolated from the *Tachypleus tridentatus* [37], and it has direct antimicrobial effects at high concentrations [38]. In a high-fat disease mouse model, kynurenic acid can inhibit the increase in Firmicutes and Bacteroidetes, which are associated with the disease. However, kynurenic acid promotes the growth of probiotics, such as *Lactobacillus acidophilus*, *Bifidobacterium* and *Actobacillus rhamnosus* [39]. Additionally, Robert et al. found that kynurenine easily enters probiotics, preferentially converts into kynurenic acid, and is then swiftly released into the extracellular environment to act [40]. Furthermore, the intestinal antimicrobial protein REG3A influences gut microbiota composition by increasing the proportion of lactobacilli, which in turn increases kynurenine levels [41]. Therefore, kynurenine and its metabolites are closely linked with the gut microbiota, working together to regulate the occurrence and development of liver diseases.

### 2.2. Biological Activity of the Kynurenine Pathway and Its Derivatives

In the metabolism of tryptophan, kynurenine synthesis is catalyzed by tryptophan-2,3-dioxygenase (TDO) and indoleamine 2,3-dioxygenase (IDO). The role of kynurenine in the immune system has been widely studied. Kynurenine and its derivatives function as agonists of the aryl hydrocarbon receptor (AhR) and serve as critical modulators of inflammatory processes [42]. AhRs are a family of proteins found in various species, including humans, and are ligand-activated transcription factors [43].

AhR signaling is considered a critical component of immune responses at barrier sites. It can influence intestinal epithelial renewal, barrier integrity, and various immune cells such as intraepithelial lymphocytes, Th17 cells, innate lymphoid cells, macrophages, dendritic cells, and neutrophils, which are essential for gut homeostasis [44]. Activated AhR signaling is closely associated with inflammatory diseases, such as ulcerative colitis [45], non-alcoholic steatohepatitis [46], and systemic lupus erythematosus [47]. This process can regulate immune cell functions through multiple pathways, including AhR-induced Cyp1a1 gene expression [48], AhR interaction with NF-κB [49], and AhR regulation of the AhR/Nrf2/NQO1 pathway [50]. The AhR signaling pathway in cells can also be activated by ligands such as Kyn and its derivatives. The kynurenine pathway represents a potential target for modulating gut microbiota in liver inflammatory diseases and plays a crucial role in the inflammatory signaling of the AhR–liver axis [51].

Kynurenine and its derivatives are potential immunosuppressive metabolites produced through the tryptophan metabolic pathway. It has been reported that kynurenine can induce immune tolerance, acting as an endogenous ligand for AhR. Elevated kynurenine levels activate the aryl hydrocarbon receptor, thereby inhibiting effector T cell proliferation [7] and inducing the FoxP3 transcription factor to promote the differentiation of immunosuppressive Treg cells. In addition, kynurenine also suppresses the surface expression of the NKp46 and NKG2D activating receptors, thereby inhibiting T cell receptor expression and NK cell function—key mechanisms implicated in immune evasion across various cancers [52]. For example, Patra et al. [46] found a positive correlation between indoleamine 2,3-dioxygenase-1 (IDO1) and the tumor-promoting reprogramming transcription factor Lin28B in HCC patient samples [53]. Lin28B can increase the production of the enhancer Sox2/Oct4 to upregulate the expression of IDO1. The effect of IDO1 inhibitors is minimal when used alone, but when combined with immune checkpoint inhibitors, the anti-tumor effect is significantly enhanced. In liver cancer cell models, inhibiting IDO1 expression can significantly enhance the immune response against the tumor and slow tumor growth. This result suggests that the role of IDO1 in tumor immune evasion should not be underestimated, and its inhibition may become a new immunotherapeutic strategy.

The role of kynurenine and its metabolites in the central nervous system has also received increasing attention. Kynurenine, through its downstream metabolites, such as kynurenic acid, 3-hydroxykynurenine, 3-hydroxyanthranilic acid, and quinolinic acid, plays an important role in neurotoxicity, neuroinflammation, and the development of neurodegenerative diseases. Kynurenine aminotransferase (KAT) can convert kynurenine into kynurenic acid (KYNA) [54], while KMO converts kynurenine into 3-hydroxykynurenine (3-HK) [20]. Kynurenic acid (KYNA) inhibits the N-methyl-D-aspartate receptor (NMDAR) [55,56], binds to the G protein-coupled receptor 35 (GPR35), [57] and activates the aryl hydrocarbon receptor (AhR) [58], exerting anti-inflammatory, neuroprotective, and immune-regulatory effects. Conversely, the downstream metabolites of kynurenic acid, 3-hydroxykynurenine, and quinolinic acid exhibit opposite effects. 3-Hydroxykynurenine is a toxic metabolite that induces oxidative damage and cell death [59]. Quinolinic acid acts as an NMDAR agonist, leading to excitotoxicity [60]. Alterations in the kynurenine metabolic pathway are considered crucial in the pathological processes of neurodegenerative diseases such as Alzheimer’s disease (AD), Parkinson’s disease (PD), and Huntington’s disease (HD). A clinical study found that in Parkinson’s disease (PD) patients, the levels of kynurenine metabolite 3-hydroxykynurenine (3-HK) were significantly elevated in both peripheral and central systems, and this elevation correlated with the severity of the disease [61]. The kynurenine metabolic pathway not only plays a significant role in neurological diseases but is also closely related to the occurrence of psychiatric disorders [62]. The potential mechanism of elevated KYNA in psychiatric disorders may be related to increased activation of the pro-inflammatory cytokine IL-1β. Kynurenic acid, as an NMDA receptor antagonist, is elevated in the cerebrospinal fluid of patients with schizophrenia and mania [63]. Moreover, central nervous inflammation may have an important relationship with the activation of the kynurenine pathway in mental illnesses [64,65]. In conclusion, inhibiting the kynurenine metabolic pathway is considered a potential therapeutic strategy, with inhibition of IDO1 activity being an important target for neuroprotective treatments to alleviate symptoms of neurodegenerative diseases and neuroinflammation.

Kynurenine can affect liver cell survival and death by regulating apoptosis pathways. During liver injury or disease progression, kynurenine may affect liver health by promoting T cell apoptosis in the liver [66] or inhibiting cell proliferation. The immune tolerance of the liver, the metabolic status of liver cells, and the local inflammatory response are closely related to the metabolism of kynurenine. Liver injury is often accompanied by immune system activation and inflammatory responses. Therefore, kynurenine prevents liver injury by alleviating oxidative stress, inflammation, and cell death. In acute liver injury, kynurenine can suppress T cell proliferation via apoptosis pathways, promoting immunosuppressive responses and thus slowing liver recovery [67]. Moreover, the kynurenine metabolite 3-hydroxyanthranilic acid accumulates in monocytes/macrophages, thereby inducing immune cell apoptosis [68]. Bishnupuri et al. found that in IDO1 knockout mouse colon tumor model, kynurenine pathway metabolites rapidly activated PI3K-Akt signaling in tumor epithelial cells and promoted β-catenin nuclear translocation, cell proliferation, and anti-apoptotic effects [69].

## 3. The Role of Kynurenine and Its Derivatives in Liver Diseases

The roles of kynurenine and its derivatives in liver diseases are complex and multifaceted. They may play an important role in the occurrence and development of various liver diseases, such as liver injury, liver inflammation, liver fibrosis, and liver cancer, by regulating immune responses, oxidative stress, apoptosis, fibrosis, and other processes. Kynurenine and its derivatives cannot only regulate diseases through various mechanisms, but their metabolite levels are also closely related to the occurrence and development of diseases (Table 1). Therefore, kynurenine and its metabolites not only provide potential biomarkers for early diagnosis of liver diseases [70], but also offer possible new therapeutic targets. This section discusses reprogramming of the kynurenine metabolism, the abnormal expression of key metabolic enzymes and their products in various liver diseases, and their potential clinical significance and prognostic value.

### 3.1. Acute Liver Injury

Acute liver injury (ALI) is an acute inflammatory response of the liver caused by factors such as drugs, viral infections, alcohol intoxication, and ischemia–reperfusion, characterized by hepatocellular degeneration, necrosis, and apoptosis, and is a multi-etiologic clinical syndrome [87]. Hepatocellular death triggers subsequent inflammation, leading to excessive deposition of extracellular matrix proteins. Therefore, liver injury often leads to hepatitis and liver fibrosis, which are important initiating factors for cirrhosis and hepatocellular carcinoma (HCC).

**Figure 3:**
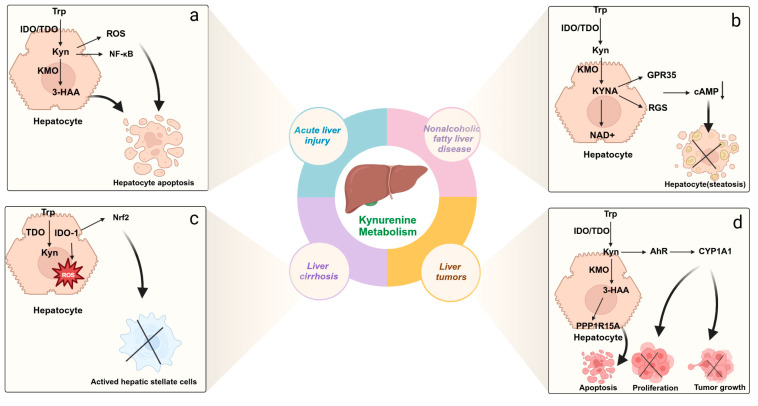
Molecular mechanisms of kynurenine pathway in liver diseases. (**a**) In acute liver injury, the metabolic migration of kynurenine pathway from kynurenine (Kyn) to nicotinamide adenine dinucleotide leads to endoplasmic reticulum stress and activation of NF-κB signaling pathway to induce hepatocyte apoptosis. (**b**) In metabolic dysfunction-associated steatotic liver disease, the kynurenine pathway activates Gpr35 and RGS to increase energy expenditure, regulate fatty acid metabolism, and improve inflammation. (**c**) In liver cirrhosis, IDO-1 can inhibit hepatic stellate cell activation and scavenge free radicals to reduce oxidative damage by decreasing nuclear factor E2-related factor 2 (Nrf2). (**d**) The kynurenine pathway activates AHR, thereby inhibiting tumor initiation and progression and promoting tumor cell apoptosis. Abbreviations: Trp, tryptophan; KYN, kynurenine; KYNA, kynurenic acid; 3-HAA, 3-hydroxyanthranilic acid; ROS, reactive oxygen species; NF-κB, nuclear factor-κB; NAD+, nicotinamide adenine dinucleotide; IDO, indoleamine 2,3-dioxygenase; TDO, tryptophan 2,3-dioxygenase; KMO, kynurenine 3-monooxygenase; RGS, regulator of G protein; GPR35, G protein-coupled receptor 35; AhR, aryl hydrocarbon receptor; CYP1A1, cytochrome P450, family 1, subfamily A, polypeptide 1.

Recently, studies have shown that in acute liver injury caused by liver ischemia–reperfusion, the kynurenine metabolic pathway undergoes reprogramming, with a decrease in liver 3-hydroxyanthranilic acid (3-HAA) and quinolinic acid (QA), while kynurenine and kynurenic acid (KYNA) levels increase. This is due to the significant upregulation of kynurenine aminotransferase 2 in hepatocytes, shifting the kynurenine pathway from 3-HAA and QA to KYNA and NAD synthesis, alleviating oxidative stress, inflammation, and cell death in hepatocytes [73]. Another study also showed that in a mouse liver injury model induced by hexafluoropropylene oxide dimer acid (HFPO-DA), the correlation between kynurenic acid (KYNA) levels in mouse serum and liver injury was the highest. Exposure to HFPO-DA-induced liver injury in mice, which caused a shift in the kynurenine pathway from kynurenic acid (KYNA) to NAD synthesis, led to endoplasmic reticulum stress and activation of the NF-κB signaling pathway (Figure 3a). Pre-treatment with kynurenic acid (KYNA) significantly alleviated liver injury induced by HFPO-DA exposure in mice, showing protective effects [88]. Hoshi et al. found that in a CCl4-induced acute liver injury mouse model with Ido2 knockout (Ido2(−/−)) and treatment with the Ido2 inhibitor 1-methyltryptophan (D-1MT), hepatocellular damage was alleviated [71]. Additionally, endothelial cells are closely related to liver function, and under the stimulation of inflammatory factors, endothelial cells highly express indoleamine 2,3-dioxygenase (IDO), which metabolizes tryptophan into kynurenine, resulting in relaxation of arterial blood vessels [89]. Kynurenine metabolism is one of the major contributing mechanisms for systemic circulatory dysfunction in acute liver failure. The increase in IDO activity, kynurenine pathway activity, and oxidative stress in endothelial cells, along with kynurenine-mediated small arterial vasodilation, represents a malignant pathological cycle, which may lead to progressive endothelial cell apoptosis [90], microcirculatory dysfunction, and organ failure. Inhibiting the kynurenine pathway can reduce hepatocellular damage and liver fibrosis. This suggests that changes in the kynurenine metabolic pathway play a key role in liver injury. However, a study by Ma et al. found that another key enzyme in the kynurenine pathway, indoleamine 2,3-dioxygenase 1 (IDO-1), increases hepatocyte ferroptosis and M1 polarization, while also enhancing M2 polarization and promoting macrophage phagocytosis [74]. Therefore, the activation of IDO-1 in macrophages plays a critical role in triggering hepatocyte death during liver ischemia–reperfusion injury.

In conclusion, kynurenine plays a role in acute liver injury through metabolic reprogramming, regulating microcirculation, and affecting NAD homeostasis, while also demonstrating some protective effects. Therefore, elucidating the role of kynurenine and its metabolites in the molecular pathology of acute liver injury is essential for understanding the connection between kynurenine metabolism and liver diseases. These findings provide new directions for future research and may help develop new therapeutic strategies to combat liver injury.

### 3.2. Metabolic Dysfunction-Associated Steatotic Liver Disease

Currently, metabolic dysfunction-associated steatotic liver disease (MASLD) is one of the leading causes of chronic liver disease worldwide [91], and the progression from non-alcoholic fatty liver to non-alcoholic steatohepatitis has become a major threat leading to cirrhosis, acute-on-chronic liver failure, and liver cancer. Among these, the kynurenine metabolic pathway plays a crucial role in the progression of non-alcoholic fatty liver disease. Dorochow et al. analyzed the liver lipidome, metabolome, and immune cells in non-alcoholic hepatitis and found that kynurenine levels were elevated in the liver. This elevation promoted M2-like macrophage polarization and inhibited the activity of natural killer cells, dendritic cells, monocytes, and macrophages, exerting an anti-inflammatory effect and slowing disease progression [76]. Kynurenic acid increases energy expenditure, improves energy metabolism, and reduces inflammation in mice on a high-fat diet by activating Gpr35 and RGS (Figure 3b) [77]. Berge et al. constructed a mouse model of non-alcoholic fatty liver and found that although kynurenine levels decreased in the plasma, the end product of kynurenine metabolism, niacinamide, increased. This change is likely unrelated to increased inflammation and oxidative stress. The increase in NAD+ and niacinamide is likely generated through the salvage pathway, rather than through the de novo kynurenine pathway [75]. Carine et al. found that treatment with 1-triple TTA in male Wistar rats resulted in increased fatty acid oxidation in liver mitochondria and promoted the conversion of tryptophan to NAD+. Therefore, kynurenine and its derivatives are positively correlated with mitochondrial fatty acid oxidation [92]. Agudelo et al. found that treatment with KYNA reduced the levels of quinolinic acid (QA) and 3-hydroxykynurenine (3-HK) in mice while increasing the levels of nicotinic acid [93]. NAD+, as the end product of tryptophan–kynurenine axis de novo synthesis, has therapeutic effects in fatty liver disease [94]. Fatty liver disease is closely associated with metabolic dysfunction, particularly with the increase in obesity. Carmen Arto et al. quantified the in vivo levels of 15 tryptophan-related metabolites in the kynurenine, indole, and serotonin pathways using ultra-high-performance liquid chromatography, and found that only kynurenine-related metabolites were associated with increased gene expression of the tryptophan degradation pathway enzymes, IDO-1 and kynurenine monooxygenase (KMO), as well as elevated concentrations of various kynurenine-related metabolites [95]. Targeting these pathways may have potential therapeutic applications.

In conclusion, kynurenine metabolism presents a pathway with potential for controlling energy homeostasis in the treatment of metabolic dysfunction–associated steatotic liver disease (MASLD). Further understanding of kynurenine and its metabolites in metabolic dysfunction-associated steatotic liver disease (MASLD) is crucial for elucidating the relationship between liver diseases.

### 3.3. Chronic Liver Injury and Liver Cirrhosis

Chronic liver injury is caused by long-term conditions, such as chronic hepatitis, fatty liver, or alcoholic liver disease, and can eventually develop into liver fibrosis or cirrhosis. Kynurenine and its metabolites play a crucial role in the formation of liver fibrosis, particularly in inhibiting the liver’s repair process through immune escape mechanisms. The pathogenesis of liver fibrosis is closely related to the immune microenvironment, with macrophages playing a key role as important immune cells in the liver, capable of shifting between two polarized phenotypes, classical (M1) and alternative (M2), in response to external stimuli [96]. Indoleamine 2,3-dioxygenase 1 (IDO1) can be detected in macrophages and it regulates various immune responses and plays a regulatory role in dendritic cells. IDO1 impairs the maturation of dendritic cells in wild-type (WT) mice and downregulates key regulators of cellular oxidative stress and inflammation, including nuclear factor E2-related factor 2 (Figure 3c), thus alleviating liver fibrosis [79]. Studies have shown that kynurenine participates in regulating immune tolerance in chronic liver injury via the IDO1 pathway. Wang et al. discovered that activation of IDO1 in a mouse model of liver fibrosis caused an increase in kynurenine levels and influenced macrophage differentiation, thereby delaying immune response recovery. Further analysis indicated that IDO1-mediated kynurenine increased the activation of hepatic stellate cells, thus promoting the progression of liver fibrosis [81]. The role of IDO-1 in liver diseases is not only related to the regulation of immune responses and fibrosis but also involves various physiological processes such as liver regeneration, oxidative stress, and inflammation. Due to the liver’s excellent regenerative capacity, especially after liver injury, hepatocytes can rapidly recover and repair. However, chronic liver diseases, such as cirrhosis, impair liver regeneration due to persistent inflammation and fibrosis. In this process, MSCs can migrate to the damaged tissue, undergo hepatocyte differentiation [97], inhibit the release of inflammatory factors, and enhance hepatocyte proliferation in the body [98]. Additionally, MSCs can secrete indoleamine 2,3-dioxygenase (IDO) to exert immune-regulatory effects [99]. Treatment with IDO1 inhibitors significantly alleviated liver fibrosis, suggesting that kynurenine metabolism plays a critical role in chronic liver injury and fibrosis.

In addition, tryptophan 2,3-dioxygenase (TDO) is another systemic tryptophan-metabolizing enzyme, and its high expression in liver is also associated with the progression of liver fibrosis [82]. Zhong et al. found in a mouse liver fibrosis model that liver lesions correlated positively with serum IDO1 levels, and IDO1 in vivo deficiency led to compensatory increases [100]. TDO exacerbates the liver’s inflammatory response and immune suppression by enhancing tryptophan metabolism to kynurenine [80]. Inhibition of TDO expression improves the degree of liver injury, indicating that TDO-mediated kynurenine metabolism is closely related to the occurrence of chronic liver injury and liver fibrosis. In chronic liver injury, the reprogramming of the kynurenine metabolic pathway is tightly regulated. Therefore, these findings highlight the role of IDO1 and TDO activity in the pathology of liver fibrosis.

### 3.4. Hepatocellular Carcinoma

Hepatocellular carcinoma (HCC) is one of the most lethal malignant tumors worldwide and can arise from various chronic liver diseases and cirrhosis. Hepatocellular carcinoma (HCC) is a highly invasive liver tumor with a pronounced capacity for metastasis to distant organs, including the lungs, bone, brain, lymph nodes, and adrenal glands. As a malignant tumor, early detection with high precision and the ability to assess the patient’s response to treatment are crucial for the prognosis. The kynurenine pathway’s catabolism plays an immunosuppressive role in HCC and enhances tumor survival and invasiveness [101]. The key enzyme of the kynurenine pathway, TDO, suppresses the tumor immune response by inducing immune tolerance, thus promoting immune escape of the tumor. TDO expression is typically elevated in the HCC microenvironment, accompanied by kynurenine accumulation, which may promote tumor growth and metastasis by affecting immune cell function in the tumor microenvironment [102]. Inhibition of TDO expression diminishes kynurenine production and inactivates the aryl hydrocarbon receptor (AhR), thereby promoting the activation and proliferation of CD3(+) T cells, which contribute to anti-tumor immune responses [84]. TDO is not only involved in immune tolerance but is also closely associated with the metastasis of HCC cells. The zinc-finger transcription factor, ZNF165, which is highly expressed in liver tissue and the immune microenvironment, can activate the kynurenine/AhR/CYP1A1 axis and promote CYP1A1 expression, thus enhancing HCC cell proliferation and migration (Figure 3d). Miyazaki et al. conducted a systematic analysis of metabolites in colon cancer patient samples and found that high levels of kynurenine and TDO2 were positively correlated with liver metastasis [103]. In a mouse colon cancer model, TDO expression significantly enhanced liver metastasis, primarily by inducing AHR-mediated PD-L1 transactivation, which suppressed the immune response and promoted liver metastasis of colon cancer. Additionally, in terms of treatment, a new immunosuppressive agent can improve the effectiveness of tumor chemotherapy. The conjugate obtained by combining it with irinotecan can improve the tumor immune microenvironment by inhibiting TDO enzyme expression to block kynurenine production and induce HCC cell apoptosis by releasing TDO inhibitors and irinotecan to cause DNA damage [85]. Kynurenine metabolic derivatives also play a role in HCC treatment. In a liver cancer nude mouse model, 3-HAA was found to increase apoptosis of HCC in cultured cells and mouse xenografts by upregulating phosphatases PPP1R15A/DUSP6 and reducing AKT phosphorylation, as well as increasing sensitivity to the first-line targeted drug sorafenib in HCC patients [83]. It has been reported that high serum kynurenine levels are associated with poor prognosis in HCC. Bekki et al. demonstrated that elevated kynurenine levels in the serum of HCC patients may serve as a biomarker for predicting survival and prognosis in individuals with early-stage HCC [104]. In conclusion, the kynurenine pathway plays an important role in immune escape and tumor progression in HCC. Elucidating these pathways will open new avenues for therapeutic strategies targeting kynurenine metabolites in liver cancer.

## 4. Clinical Applications of the Kynurenine Pathway

The kynurenine pathway plays an important role in human health and disease, and the regulation of kynurenine metabolism has clinical and therapeutic significance. Currently, the primary clinical treatments include inhibitors targeting different enzymes in the kynurenine pathway, such as IDO1 inhibitors, TDO inhibitors, and KMO inhibitors, which have shown efficacy in cancer, immune diseases, neurological diseases, and neurodegenerative diseases (Table 2).

Currently, significant exploration of IDO1 inhibitors has shown promising progress in clinical settings, including drugs such as Epacadostat, BMS-986205, 1-methyl-tryptophan (1-MT), and EOS200271 (formerly PF-06840003). Epacadostat is a tryptophan competitive inhibitor with high selectivity for IDO1. Animal studies have shown that Epacadostat can reduce tumor growth and promote the proliferation of T cells and NK cells [114]. In clinical applications, a phase 1/2 ECHO-203 (NCT02318277) study evaluated the combination of IDO1 inhibitor Epacadostat with Durvalumab in treating adult patients with advanced solid tumors, including melanoma (n = 5), non-small cell lung cancer (n = 20), squamous cell carcinoma of the head and neck (n = 27), and bladder cancer (n = 19). The objective response rate (ORR) was greater than 10%, with melanoma patients showing the best objective response rate, reaching 80% [105]. BMS-986205 is an oral IDO1 inhibitor. The combination of BMS-986205 with Nivolumab can improve patient response rates. For example, in bladder cancer patients (n = 25), the objective response rate (ORR) was 32%, and in cervical cancer patients (n = 22), the ORR was 14% [106]. Indoximod, as a competitive IDO1 inhibitor, can modulate the function of T cells, CD4 T cells, and dendritic cells (DCs) [113,115], while reverse IDO activity exerts immune suppression. A single-arm phase II clinical trial demonstrated promising safety and efficacy with the combination of Indoximod and Pembrolizumab. Among 89 non-ocular melanoma patients, the objective response rate (ORR) was 51%, and the confirmed complete response rate was 20% [107]. PF-06840003, due to its ability to cross the blood–brain barrier (BBB), was studied in a phase I trial by David et al. in patients with recurrent malignant glioma (NCT02764151). Eight patients (47%) showed controlled disease, indicating its pharmacodynamic effects and sustained clinical efficacy in recurrent malignant glioma patients [108]. TDO, as another key enzyme in the kynurenine pathway, also holds immense potential in clinical treatment. TDO inhibitors include 680C91, LM10, and others. In a preclinical study, Tsai-Der Chuang et al. found that treatment with 680C91 in immunodeficient mice bearing human fibrosarcoma xenografts reduced the weight of uterine fibroid xenografts by 30% [109] S. Hu et al. discovered that in a squamous cell carcinoma mouse model, the TDO2 inhibitor LM10 alleviated T cell suppression, restored T cell anti-tumor responses, and blocked the malignant progression of squamous cell carcinoma [110].

Due to the tremendous progress in small-molecule inhibitors targeting tryptophan metabolism, especially IDO and TDO inhibitors in cancer therapy, dual-target inhibitors targeting both IDO1 and TDO are being developed for broader and more effective clinical treatments of various diseases, including M4112 and RY103. M4112, as an effective and selective dual inhibitor of IDO1 and TDO2, was shown in a tumor mouse model to possess dual inhibitory effects. It was also preliminarily validated for anti-tumor activity in solid tumors, with the best overall response being disease stabilization in 9 out of 15 patients (60.0%) [111]. Subsequently, Zhang et al. developed a new IDO1/TDO dual-target inhibitor compound 17 (RY103). In an in situ pancreatic cancer mouse model, RY103 was able to inhibit tumor growth and metastasis, promote tumor cell apoptosis, significantly improve the immune suppression state, and show no significant toxicity to the mice [112]. Additionally, KMO is overexpressed in several diseases, especially in cancers like melanoma, colorectal cancer, and breast cancer [116], as well as in neurodegenerative diseases such as Alzheimer’s disease and Huntington’s disease [117,118], contributing to disease progression. Therefore, research on KMO inhibitors shows potential as a promising therapeutic strategy for a variety of diseases. Zwilling et al. discovered that JM6 is a small-molecule prodrug inhibitor of kynurenine 3-monooxygenase (KMO) that can inhibit KMO in the blood. In a transgenic mouse model of Alzheimer’s disease, it prevented spatial memory deficits, anxiety-related behaviors, synapse loss, and prolonged survival [113].

Inhibitors of the kynurenine pathway can exert anti-tumor effects when combined with other immunotherapies, such as immune checkpoint inhibitors, chemotherapy, and radiotherapy; however, their application in liver diseases is not yet widespread, possibly due to the complex role of IDO in the liver and the lack of sufficient clinical evidence to support their broad use in liver-related diseases. Therefore, although basic research has made some progress, indicating that kynurenine and its derivatives have antioxidant, anti-inflammatory, and pro-apoptotic effects on liver cells and are closely related to the occurrence and development of liver diseases, most studies focus on mouse models, and clinical application is still distant. However, related drugs targeting KP enzyme inhibitors have been applied in other disease treatments. A deeper understanding of kynurenine and its derivatives, as well as the rational design of IDO1 inhibitors in liver diseases, could provide new avenues for therapeutic intervention in liver diseases.

## 5. Conclusions

In summary, the kynurenine pathway and its various metabolites, such as kynurenic acid (KYNA), 3-hydroxykynurenine (3-HK), and quinolinic acid (QA), play an important role in liver diseases. Under pathological conditions, the dysregulation of metabolic enzyme activity in the kynurenine pathway, such as increased IDO expression and imbalance in metabolite levels, leads to immune activation and activation of inflammatory pathways, which play a critical role in the progression of liver diseases. With the development of multi-omics and metagenomic technologies, research on the mechanisms of the kynurenine metabolic pathway in liver diseases has deepened. The kynurenine metabolic pathway, especially the inhibitors of kynurenine pathway enzymes, has become an important target for clinical diagnosis and new drug development in liver diseases [119]. For example, 5-fluorouracil combined with indoleamine 2,3-dioxygenase (IDO) inhibitors, assembled into nanoparticles, effectively reverses drug resistance and enhances immunotherapy for liver cancer [120]. However, the selectivity, efficacy, and safety of enzyme inhibitors pose a challenge in clinical applications. Therefore, targeting the kynurenine metabolism pathway may be an important approach for treating liver diseases in the future, although most research is still in the basic research phase. In the future, conducting more research to improve the molecular mechanisms linking kynurenine metabolites to liver pathological states and achieving a comprehensive understanding of kynurenine and its derivatives will help expedite clinical translation.

## Figures and Tables

**Table 1:** Changes and roles of kynurenine and its metabolites in liver diseases.

**Liver Diseases**	**Levels of Kynureine** **Metabolites**	**Roles of Kynureine** **Metabolites**
Acute liver injury	Increased KYN and KYNA [71] Decreased 3-HAA and QA [72]	1. Modulate endothelial cells to reduce liver injury [70] 2. Reduce oxidative stress in liver cells [73] 3. Regulate immune cells, such as monocytes/macrophages, T cells, dendritic cells, NK cells, etc. [74]
Metabolic dysfunction-associated steatotic liver disease	Increased NAD+ [75] Decreased KYN [75]	1. Inhibit immune cell activity [76] 2. Promoting the activity and proliferation of regulatory T cells [76] 3. Alleviating oxidative damage in the liver [76] 4. Regulating fatty acid metabolism and lipid transport [77] 5. Promoting hepatocyte repair and regeneration [78]
Liver fibrosis and cirrhosis	Increased KYN [79]	1. Reduce cellular oxidative damage and inflammation [79,80] 2. Reducing immune–inflammatory responses [79] 3. Eliminating free radicals to reduce oxidative injury [79] 4. Suppressing the activation of hepatic stellate cell [81] 5. Correcting metabolic disturbances in the liver [82]
Liver tumors	Increased KYN and 3-HA [83]	1. Inhibit immune cell function [84] 2. Inhibit proliferation and metastasis of liver cancer cells [84] 3. Alleviating tumor-associated inflammation [84] 4. Inhibiting the tumor microenvironment [85] 5. Suppressing tumor angiogenesis [86]

Abbreviations: KYN, kynurenine; KYNA, kynurenic acid; 3-HAA, 3-hydroxyanthranilic acid; 3-HA,3-hydroxyanthranilic acid; QA, quinolinic acid; NAD+, nicotinamide adenine dinucleotide.

**Table 2:** Clinical applications of kynurenine pathway inhibitors.

Drug Name	Formulation and Dose	Mechanism of Action	Disease	Clinical Results
Epacadostat	Oral, 100 mg twice daily	IDO1 inhibitor	Melanoma	In a phase 1/2 study, ORR was 80% in melanoma patients (n = 5), with 40% of the patients achieving complete response [105].
			Bladder cancer	In a phase 1/2 study, ORR was 15.8% in bladder cancer patients (n = 19), and CRR was 5.3% [105].
BMS-986205	Oral, 100 mg once daily	IDO1 inhibitor	Bladder cancer	In a clinical study, ORR in bladder cancer patients (n = 25) was 32% [106].
			Cervical cancer	In a clinical study, ORR in cervical cancer patients (n = 22) was 14% [106].
Indoximod	Oral, 600 mg twice daily	IDO1 inhibitor	Melanoma	In a phase II trial, ORR was 51% in melanoma patients (n = 89), with 20% of the patients achieving complete response [107].
PF-06840003	Oral, 500 mg twice daily	IDO1 inhibitor	Recurrent malignant glioma	A phase I study showed 47% of patients (n = 8) had disease control, indicating sustained clinical efficacy [108].
680C91	Intraperitoneal injection, 100 mL per day	TDO inhibitor	Uterine fibroids	A preclinical study showed a 30% reduction in the weight of uterine fibroid xenografts in immunodeficient mice [109].
LM10	Oral gavage, 160 mg/kg per day	TDO inhibitor	Squamous cell carcinoma	Preclinical studies in mouse models showed T cell anti-tumor responses and prevented the malignant progression of squamous cell carcinoma [110].
M4112	Oral, 600 mg twice daily	Dual IDO1–TDO inhibitor	Solid tumors	A phase I study showed 60% of patients (n = 15) had disease control [111].
RY103	Intraperitoneal injection, 12 mg/kg every 36 h	Dual IDO1–TDO inhibitor	Pancreatic cancer	In pancreatic cancer mouse model, RY103 inhibited tumor growth, metastasis, and improved immune suppression [112].
JM6	Oral gavage, 100 mg/kg per day	KMO inhibitor	Alzheimer’s disease	In transgenic Alzheimer’s mouse models, JM6 prevented spatial memory deficits, anxiety behaviors, synapse loss, and extended survival [113].

Abbreviations: ORR, objective response rate; CRR, complete response rate.

## Abbreviations

The following abbreviations are used in this manuscript:

NAD+	Nicotinamide adenine dinucleotide
IDO	Indoleamine 2,3-dioxygenase
TDO	Tryptophan 2,3-dioxygenase
KMO	Kynurenine 3-monooxygenase
KAT	Kynurenine aminotransferases
KMO	Kynurenine 3-monooxygenase
3-HAO	3-Hydroxyanthranilate 3,4-dioxygenase
KYN	Kynurenine
KYNA	Kynurenic acid
3-HK	3-Hydrokynurenine
Trp	Tryptophan
IPA	Indole-3-propionic acid
IAA	Indole-3-acetic acid
SCFAs	Short chain fatty acids
3-HA	3-Hydroxyanthranilic acid
QA	Quinolinic acid
3-HAA	3-Hydroxyanthranilic acid
QPRT	Quinolinic acid phosphoribosyl transferase
BBB	Blood–brain barrier
KP	Kynurenine pathway
AhR	Aryl hydrocarbon receptor
ROS	Reactive oxygen species
IFN-γ	Interferon-γ
TNF-α	Tumor necrosis factor-alpha
ALI	Acute liver injury
MASLD	Metabolic dysfunction-associated steatotic liver disease
HCC	Hepatocellular carcinoma
RGS	Regulator of G protein
GPR35	G protein-coupled receptor 35
CYP1A1	Cytochrome P450, family 1, subfamily A, polypeptide 1
NMDAR	N-methyl-D-aspartate receptor
ORR	Objective response rate
CRR	Complete response rate
